# Ejaculate Oxidative Stress Is Related with Sperm DNA Fragmentation and Round Cells

**DOI:** 10.1155/2015/321901

**Published:** 2015-02-23

**Authors:** Valeria Maria Iommiello, Elena Albani, Alessandra Di Rosa, Alessandra Marras, Francesca Menduni, Giovanna Morreale, Shanti Lia Levi, Benedetta Pisano, Paolo Emanuele Levi-Setti

**Affiliations:** Humanitas Fertility Center, Department of Gynaecology, Division of Gynecology and Reproductive Medicine, Humanitas Research Hospital, Via Manzoni 56, Rozzano, 20089 Milan, Italy

## Abstract

Oxidative stress (OS) plays an essential role in male infertility aetiology by affecting sperm quality, function, and also the integrity of sperm DNA. The assessment of oxidative stress in semen may be an important tool to improve the evaluation of sperm reproductive capacity. The purpose of this study was the evaluation of any possible relation between the unbalance of oxidative stress caused by superoxide anion in the ejaculate with the presence of sperm DNA fragmentation and high concentration of round cells. 56 semen samples from males from couples suffering from infertility were evaluated according to World Health Organisation (WHO) 2010 guidelines. Oxidative stress levels from N1 (low) to N4 (high) were assessed in ejaculates using oxiSperm; DFI (sperm DNA fragmentation index) as assessed by the SCSA (Sperm Chromatin Structure Assay) was used for evaluation of sperm chromatin integrity. Our data show that high oxidative stress (N3-N4 levels) correlated positively with a DFI ≥ 30% (*P* = 0.0379) and round cells ≥1.500.000/mL (*P* = 0.0084). In conclusion, OS increases sperm DNA damage. Thus evaluation of semen OS extent of sperm DNA damage in infertile man could be useful to develop new therapeutic strategies and improve success of assisted reproduction techniques (ART).

## 1. Introduction

One of the causes of sperm DNA damage and male infertility is the presence of unbalanced reactive oxygen species (ROS) [1]. OS occurs when ROS production exceeds the body's own natural antioxidant defences, resulting in cellular damage.

There are three different forms of ROS: the primary form of ROS including superoxide anion from which secondary ROS can be derived directly or indirectly, the secondary form of ROS including hydrogen peroxide, hydroxyl radical, and peroxyl radical, and the tertiary form of ROS represented by nitrogenous compounds (peroxynitrous acid, nitroxyl anion, peroxynitrile, and nitrous oxide) [2]. Human ejaculate consists of different types of cells such as mature and immature spermatozoa, round cells at different stages of the spermatogenic process, leukocytes, and epithelial cells. The two major sources of ROS in semen are leukocytes and spermatozoa themselves. However, it is implied that leukocytes contribute the most to OS because compared with spermatozoa the rate of ROS production in leukocytes is 1000 times greater. This is considered an “extrinsic source” of ROS as opposed to the “intrinsic source” from sperm which correlates strongly with different sperm parameters such as DNA fragmentation [3–5]. In addition, OS in semen may be secondary to a lot of other exogenous sources such as environmental pollution by heavy metals and lifestyle factors such as obesity, smoking, and alcohol abuse but also some medical conditions such as varicocele, spinal cord injury, and genitor-urinary tract infections [4]. It also describes a significant increase in seminal ROS production in men older than 40 years [6]; despite these higher seminal ROS levels in older men, no increase in the seminal leukocyte concentration was found.

Low and controlled concentrations of ROS play an important role in sperm physiological processes such as capacitation, hyperactivation, acrosome reactions, and signaling processes necessary for fertilization. Moreover, an increase in OS significantly impairs sperm function; these impairments have resulted in male infertility [7].

In fact, ROS can damage sperm membrane resulting in poor motility and impaired sperm-oocyte fusion [8]. As previously described, OS can also be linked with sperm DNA damage which may result in poor embryo development, miscarriage, and infertility [9]. ROS attack the integrity of DNA in sperm nucleus resulting in base modification, strand breaks, and chromatin cross-link. Sperm chromatin has a highly condensed and organized structure that helps to protect it from oxidative damage, making it very resistant to DNA damage [10]. However, when compaction is poor and chromatin protamination is incomplete sperm DNA is more vulnerable to OS.

Several studies have pointed out a central role of oxidative stress, leading to the formation of ROS, in the etiology of sperm DNA damage [7, 11–13]. These findings extended earlier original observations on the role of oxidative stress in the etiology of male infertility [14, 15]. High ROS concentrations in infertile men have been associated with DNA fragmentation and poor chromatin packing.

Some studies have shown that the administration of antioxidant may improve sperm DNA integrity [16, 17] and pregnancy outcomes [18]; therefore, there is a clear need for andrology laboratories to be able to identify sperm OS.

Routine semen analysis has allowed clinicians to make a fairly accurate OS diagnosis. A reduction of some semen parameters is frequently seen in man with OS; also hyperviscosity of seminal plasma [19] and* Ureaplasma urealyticum* infections [20] are associated with increased ROS production. As previously described, the presence of a large number of round cells can imply OS caused by leukocytospermia [21] besides abnormal sperm morphology related to excess residual cytoplasm and cytoplasmic droplets [22].

Fertile healthy men with normal seminal parameters almost consistently have low levels of DNA breakage, while infertile men with abnormal seminal parameters have higher fraction of sperm DNA damage [23]. Idiopathic infertile men may present normal routine seminal parameters with abnormal DNA integrity [24].

The aim of our study was to evaluate any possible relationship between the unbalance of oxidative stress in human semen assessed by OxiSperm with the presence of an increased sperm DNA fragmentation estimated by Sperm Chromatin Structure Assay (SCSA) and high concentration of round cells.

## 2. Materials and Methods

The study sample included 56 randomly selected males from couples suffering from male infertility between October 2013 and February 2014. Male factor infertility is defined as the inability of a couple to conceive a child after one year of unprotected sexual intercourse with a female that has a normal reproductive history, normal ovulation, and tubal patency. Male factor infertility patients included in the study consisted of both men with normal sperm parameters according to World Health Organization (WHO) criteria [25] and men who had at least one defect in either count, motility, or morphology. Samples with severe dispermy (sperm concentration minor of 2 × 10^6^/mL) were not included; a minimum amount of sperm to reliably perform the various sperm assays is required.

### 2.1. Semen Collection and Analysis

Semen samples were produced by masturbation after a period of 2–7 days of sexual abstinence. After liquefaction at 37°C, standard comprehensive semen analysis of the evaluation of macroscopic and microscopic parameters was performed according to WHO 2010 guidelines [25]. A particular attention was used for the evaluation and estimation of round cells.

Both SCSA and OxiSperm require an extremely small fraction of the total ejaculate with a reasonable sperm count. An aliquot of semen was used for oxidative stress estimation by OxiSperm (Halotech DNA, Madrid, E, EU). For SCSA, another semen aliquot was diluted in isotonic phosphate buffered saline (PBS) for adjusting sperm concentration to 2 × 10^6^/mL and frozen in liquid nitrogen; cryoprotectants are not needed. Samples with sperm concentration minor of 5 × 10^6^/mL were diluted in proportion 1 : 1 semen-PBS.

### 2.2. Assessment of OS by OxiSperm

**Figure 1:**
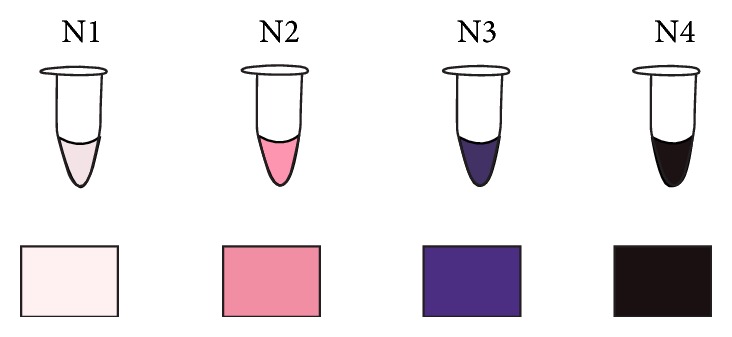
Color scheme of reactive gel intensity color (excess of superoxide anions) after reaction with superoxide anion present in the ejaculate. N1: low OS, N2: low-medium OS, N3: medium-high OS, and N4: high OS.

OS levels from N1 (low) to N4 (high) were assessed using OxiSperm kit that measures an excess of superoxide anions present in the ejaculate. The test is based on the nitro blue tetrazolium assay (NBT), a well-established laboratory technique used to quantify neutrophil function and cellular oxidative metabolism [26]. The NBT test has been shown to be useful in quantifying both leukocytes [27] and ROS production [28]. In OxiSperm kit nitro blue tetrazolium is in the form of a reactive gel (RG); water soluble tetrazolium salt incubated with cells is converted by the action of superoxide anions into an insoluble blue crystal known as formazan. These crystals are trapped within the cell but can be released by solubilization in a solvent solution; they produce and increase color intensity in the RG from yellow to different levels of purple-blue which can be easily and comparatively quantified by eye through the use of a color scale. The intensity of the color is related to the level of OS in the sample (Figure 1).

RG was liquefied in a water bath at 90°C for 5 minutes and then the RG temperature was reduced to 37°C. The RG was mixed with the semen sample in an Eppendorf tube in order to have a final sperm concentration of 1 × 10^6^/mL (volume proportion 1 : 1 semen-RG); it is possible to multiply the volumes by a common factor up to a maximum of 100 *μ*L for both of the parts of the mix. A temperature close to 37°C must be maintained when mixing the semen sample with the RG; otherwise the mix will jellify. The mix was gelified at 4°C for 5 minutes and then incubated for 45 minutes at 37°C. The resulting color of the mix was compared with the color scheme and the corresponding OS level was estimated. Alternatively a colorimeter can be used to measure the absorbance of wavelengths ranging from 530 nm to 630 nm.

To obtain the best results, the test was done using fresh semen samples and just after liquefaction. The samples were tested not after 60 minutes after ejaculation; false positives may be obtained if longer delays are produced.

It is also very important to have a precise count of sperm concentration. In effect, high sperm concentrations may produce high color intensities because of acceleration of OS pressure by sperm collision followed by membrane damage.

Samples were subsequently divided into two groups depending only on the OS level detected with OxiSperm: low OS samples (LOS) including levels N1 and N2 and high OS samples (HOS) including levels N3 and N4. LOS samples have optimal/low levels of ROS considered not able to damage cells. HOS samples have levels of ROS so high that may cause pathological effects on sperms such as DNA fragmentation.

### 2.3. Assessment of Sperm DNA Fragmentation by SCSA

Sperm DNA damage was assessed by SCSA as previously described. This is a flow cytometric test that measures the ability of sperm DNA to maintain its native double-stranded form after exposure to an acid environment, followed by staining with metachromatic fluorescent dye acridine orange (AO) (6 mg/mL Sigma, St. Louis, MO, USA) to differentiate single-stranded and double-stranded DNA. Sperms with damaged DNA denature more easily than sperms with intact DNA. AO intercalates into native DNA and fluoresces green when exposed to blue light and fluoresces red when associated with single-stranded (abnormal) DNA.

The percentage of sperms with denatured DNA was initially termed the percentage* Comp alpha t* (cells outside the main population) and has recently changed to* DNA fragmentation index (DFI)*. The assay measures the green and red fluorescence emitted by each individual sperm. The term* alpha t* is created from these fluorescence values, which uniquely identifies each sperm based on its own metachromatic emission characteristics.

SCSA was carried out following the protocol described by Evenson et al. [29] with some modification of our laboratory.

Flow cytometer* Beckman Coulter FC 500 *(Beckman Coulter, Pasadena, CA, USA) was set up with excitation at 488 nm filters and dichroic filters to collect red (≥630 nm) and green (515 to 530 nm) fluorescence.

Frozen samples were rapidly thawed in a 37°C water bath until the last remnant of ice disappears and 100 *μ*L of sample was treated with 200 *μ*L of acid solution (0.08 N HCl, 0.15 M NaCl, and 0.1% Triton-X 100, pH 1.2) for 30 seconds to induce sperm DNA denaturation. 600 *μ*L of AO staining solution (1.2 mL, 6 *μ*g/mL) was added to sperm suspension and incubated for 3 minutes. The stained sample was placed on the flow cytometer and sample flow was started immediately.

10.000–20.000 cells were analyzed in each sample. Duplicate measurements were performed taking the sample from the same thawed aliquot in order to have statistical considerations.

**Figure 2:**
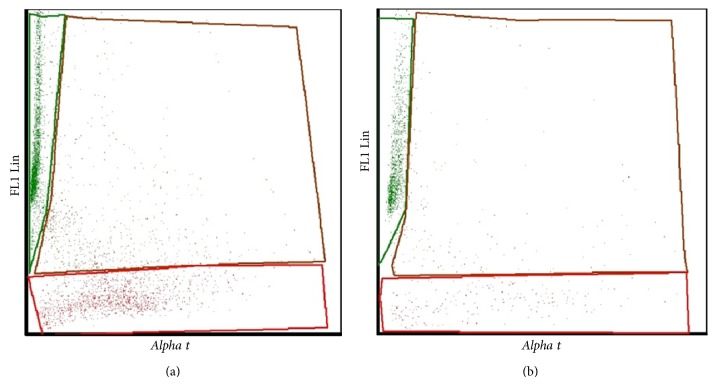
Scatter plots of SCSA analysis of a sperm sample with DFI ≥ 30% (a) and DFI < 30% (b) showing the ratio of green (not fragmented) and red (fragmented) sperm. The percentage of sperm with denatured DNA is reported as* Comp alpha t* (cells outside the main population).

The percentage of sperms with denatured DNA was expressed in terms of DFI using* FCS4 Express* software that creates a scatter plot showing the ratio of green (not fragmented) and red (fragmented) sperms (Figure 2). A statistical threshold has been established to <30% and ≥30% DFI for normal and high sperm DNA fragmentation, respectively.

### 2.4. Statistical Analysis

Data were presented as median values (interquartile ranges) as sperm parameters were not normally distributed. Two-sample Wilcoxon rank-sum (Mann-Whitney) test was used to test the relationship between OS level and sperm DNA damage (% DFI) and between OS level and semen parameters including concentration of round cells. *P* value <0.05 was considered statistically significant.

A linear regression analysis was also done to address the change of DFI in accordance with the increase of OS level.

## 3. Results

Table 1 shows *P* value of the data from seminal analysis of the samples analyzed in the study. No statistically significant differences were found (*P* < 0.05) when comparing the values obtained for each of the conventional semen analysis variables between the two groups. The parameters evaluated in seminal analysis included sperm concentration (*P* = 0.2297), sperm motility (*P* = 0.9462), sperm viability (*P* = 0.7312), and the percentage of normal sperm forms (*P* = 0.5513).

The median of DFI and round cells concentration in the two groups is reported in Table 2.

Regarding the assessment of the integrity of sperm DNA we evidenced a higher and statistically more significant DFI (DFI ≥ 30%) in the HOS group as compared to the LOS group (*P* = 0.0379) suggesting that superoxide anion unbalance is related to sperm DNA fragmentation. HOS group shows a DFI median of 21.58% (6.84–64.44) while the LOS group has a DFI median of 16.345% (4.99–45.07). The linear regression analysis shows a statistically significant relationship between DFI and OS level with a correlation coefficient value of 3.76 (95% CI: 0.52, 6.99).

By evaluating the concentration of round cells in semen samples, we observed high levels of OS in the presence of high concentration of this kind of cells suggesting a strong correlation between round cells concentration >1.5 × 10^6^/mL in semen and high OS levels (*P* = 0.0084); this supports the knowledge that ROS in human ejaculate originate mainly from leukocytes. The median concentration of round cells in HOS semen group is 0.8 × 10^6^/mL (0.1–3.3) in opposition to 0.3 × 10^6^/mL (0.1–2.5) in the LOS group.

## 4. Discussion

OS has been identified as one of the main causes of male infertility by causing sperm dysfunction. OS is a state related to increased cellular damage triggered oxygen and oxygen-derived free radicals known as ROS [30].

Usually, most clinicians do not test infertile man for OS presence because available tests are expensive or difficult to perform as compared to a routine semen analysis. As antioxidant supplementation may improve sperm DNA integrity and pregnancy outcome, there is a clinical need to perform assays to identify OS in order to put these results in relationship with sperm DNA fragmentation [31]. Somehow, none of the parameters recommended by WHO (sperm concentration, motility, and morphology) are sufficient for male infertility determination. Even though standard semen analysis continues to be the backbone of clinical evaluation of male infertility, WHO parameters only address few aspects of sperm quality and function and this can explain why the discriminative power in relation to fertility is quite low and why high numbers of idiopathic infertility are observed [32, 33].

To the best of our knowledge, this study is the first to correlate seminal OS using the semiquantitative test OxiSperm with markers of sperm quality such as sperm DNA fragmentation assessed by SCSA. A significant positive correlation (*P* < 0.05) between high semen OS levels depending on superoxide anion unbalance and an increase in sperm DNA fragmentation was noted, in accordance with the well-established ROS capacity to damage sperm DNA [34]. Some authors have reported a positive correlation between ROS production and sperm DNA fragmentation [4, 35]. A positive correlation (*P* < 0.05) between round cells concentration in semen major of 1.500.000/mL and growing in sperm DNA fragmentation was also noted; this is because round cells in semen include leukocytes [36] and male germ cells that are considered the main endogenous sources of ROS.

However, the relationship of high OS levels and a decrease in normal semen parameters did not reach statistical significance (*P* > 0.05) disagreeing with previous findings that spermatozoa from patients with abnormal sperm concentration, morphology, and motility show increased levels of DNA damage [13]. This is probably due to the fact that all the patients involved in the study had andrological indication for the assessment of sperm DNA fragmentation; in this class of patients are included men with unexplained infertility that is diagnosed whenever routine testing methods including semen analysis fail to identify a reason for delay in achieving pregnancy. Other testing methods such as assessment of ROS and DNA fragmentation may reveal the actual cause of infertility in these cases [37].

For these reasons an accurate assessment of the seminal OS levels should become an integral part of the andrology workup in order to assist clinicians in elucidating the fertility status and in establishing the optimal treatment. This study provides support for OxiSperm as a low cost and easy-to-perform assessment of sperm oxidative stress. We acknowledge that our study is relatively small and needs to be replicated by others and compared with other methods for the assessment of sperm DNA damage such as COMET and TUNEL assays.

The future goal will be the collection of more data in order to assess if there is a primary role of seminal plasma in the oxidative stress process as seen from the results.

## 5. Conclusion

In conclusion, OS is related to sperm DNA fragmentation and high concentrations of round cells. The evaluation of OS and sperm DNA damage would make a solid contribution to standard seminal analysis profile and become diagnostic tools for evaluation of male infertility especially of idiopathic origin which must be taken into consideration during andrological workup.

## Figures and Tables

**Table 1:** Summary statistics of semen parameters and OS level.

Parameters	*P* values
Sperm concentration (10^6^/mL)	0.2297
Sperm motility (%)	0.9462
Viability (%)	0.7312
Normal sperm forms (%)	0.5513

**Table 2:** Summary statistics of DFI and round cells concentration related to OS level.

	LOS (*n* = 27) median (min–max)	Study groups HOS (*n* = 29) median (min–max)	*P* value
DFI (%)	16.345 (4.99–45.07)	21.58 (6.84–64.44)	0.0379
Round cells (10^6^ cells/mL)	0.3 (0.1–2.5)	0.8 (0.1–3.3)	0.0084

Data are expressed as medians (interquartile range). Two-sample Wilcoxon rank-sum (Mann-Whitney) test was used for statistical analysis. *P* values represent statistical comparison between median of LOS and HOS group.
